# Intratumor microbiome-derived butyrate promotes chemo-resistance in colorectal cancer

**DOI:** 10.3389/fphar.2024.1510851

**Published:** 2025-01-15

**Authors:** Linsheng Xu, Bingde Hu, Jingli He, Xin Fu, Na Liu

**Affiliations:** ^1^ Department of Gastroenterology, The Second Affiliated Hospital of Xi’an Jiaotong University, Xi’an, China; ^2^ Department of Gastroenterology, Anqing 116 Hospital, Anqing, China

**Keywords:** colorectal cancer, chemoresistance, microbiome, butyrate, biomarkers

## Abstract

**Introduction:**

Colorectal cancer (CRC) is a leading cause of cancer-related mortality globally. Although tumor immunotherapy is widely recognized for treating unresectable CRC, challenges such as ineffective immunotherapy and drug resistance remain prevalent. While intratumor microbiome-derived butyrate has been implicated in promoting lung cancer metastasis, its role in CRC chemoresistance is not well understood. This study aimed to explore the relationship between intratumor butyrate and chemoresistance in CRC.

**Methods:**

We performed a comprehensive analysis of the microbiome composition in CRC patients with varying resistance-free survival (RFS) durations, utilizing 16S rRNA sequencing. Furthermore, we assessed the prognostic significance of circulating microbiome DNA (cmDNA) and examined the effects of exogenous butyrate supplementation on the chemosensitivity of CRC cell lines.

**Results:**

Our 16S sequencing analysis revealed a reduction in microbial diversity within tumor samples of patients with resistance, as indicated by metrics such as observed taxonomic units, Shannon, and Simpson indices. Notably, Roseburia and Fusobacteria emerged as prominent biomarkers for the resistance group, whereas Bifidobacterium, *Helicobacter*, and Akkermansia were identified as biomarkers for the non-resistant group. Utilizing a Lasso regression model, we identified six genera-Roseburia, *Helicobacter*, Gardnerella, Flavonifractor, Coprococcus, and Anaerostipes-that significantly correlated with recurrence-free survival. Furthermore, both the intratumor microbiome signature and circulating microbiome DNA were effective in accurately predicting CRC resistance. Experimental assays, including CCK8 and wound-healing, demonstrated that intratumor microbiome-derived butyrate enhances the proliferation and migration of HCT15 cells in a time- and concentration-dependent manner. Cell survival analysis further indicated that butyrate treatment significantly increased the IC50 value, suggesting heightened drug resistance in HCT15 cells. Mechanistically, this resistance was attributed to butyrate’s activation of the PI3K-AKT signaling pathway.

**Conclusion:**

Our results suggest that intratumor microbiome-derived butyrate contributes to chemoresistance in colorectal cancer, highlighting the potential prognostic and therapeutic significance of the intratumor microbiome.

## 1 Introduction

Colorectal cancer (CRC) has ascended to prominence as an aggressive and life-threatening malignancy, presenting formidable challenges to both patient survival and therapeutic efficacy (Li et al., 2024a). It currently ranks as the third most frequently diagnosed cancer globally and exists as the fourth leading cause of cancer-related mortality worldwide, with its incidence predicted to escalate by 60% by 2030 (Ferlay et al., 2013). The primary therapeutic modalities for CRC currently entail a combination of surgical intervention and chemotherapeutic intervention, wherein 5-fluorouracil (5-FU) emerging as the predominant pharmaceutical agent in the majority of treatment regimens (Li et al., 2024c). Notably, the emergence of drug resistance, particularly pertaining to 5-FU and docetaxel, which are widely acknowledged as conventional therapeutic modalities, has contributed to a progressive decline in the efficacy of curative interventions (Vodenkova et al., 2020). Furthermore, it is noteworthy that the administration of 5-FU may perturb the composition of the gastrointestinal microbiota, thereby compromising gut barrier integrity and promoting an inflammatory environment within the colon (Cai et al., 2021; Ren et al., 2024). Consequently, there arises an imperative to identify novel molecular targets to overcome treatment resistance and prolong patient survival rates.

The aberrant microbiome has emerged as a novel hallmark of cancer, intricately entwined with the multifaceted processes of cancer progression. The biological role of specific bacteria within this context is often highly context-dependent (Ma et al., 2024; Li et al., 2024b). Increasing evidence implicates intestinal microenvironmental dysfunction as being intimately associated with CRC development. Perturbations in the composition and relative abundance of the gut microbiota can disrupt its balance and homeostasis, precipitating alterations in intestinal barrier function (Wong and Yu, 2023; Wong and Yu, 2019). Notably, a symbiotic interaction exists between the gut microbiota and CRC, as evidenced by sequencing studies that have uncovered shifts in microbial composition and ecological dynamics in CRC patients. For instance, *Fusobacterium* has been reported to be enriched in lesions and stools of individuals with CRC (Yachida et al., 2019; Wang et al., 2022). *Fusobacterium* has also been demonstrated to be implicated in the chemoresistance of CRC patients by modulating innate immune signaling pathways (Yu et al., 2017). Gut microbial dysbiosis may foster tumorigenesis and progression, while specific alterations in microbial species or their metabolites may be intricately connected to tumor resistance, suggesting a promising role for microbiome-based diagnostics in clinical administration (Wong and Yu, 2019; Allen-Vercoe and Coburn, 2020; Saus et al., 2019).

Butyrate, a short-chain fatty acids (SCFAs) derivative, emerges as a pivotal metabolite generated through the enzymatic breakdown of dietary fiber by the intestinal microflora (Sanna et al., 2019; Guo et al., 2022b). Butyrate not only exerts a multifaceted role in the regulation of intestinal function, conferring a protective effect on intestinal epithelial cells, but also modulates the microbial milieu composition, serving as an inflammation inhibitor, thereby preserving the intestinal environmental equilibrium (Wang et al., 2023). Notably, studies have illustrated a multilayered association between butyrate and CRC resistance, suggesting its potential as a significant therapeutic target in CRC treatment (Luo et al., 2023; Smith et al., 2013). A recent integrated metagenomic and metabolomic analysis has revealed a decrease in butyrate-producing bacteria in CRC patients, accompanied by diminished acetate levels, implying that fecal butyrate levels could serve as a promising biomarker for assessing CRC risk or as an early indicator of disease initiation, progression, and severity (Kong et al., 2023; Jia et al., 2024). Additionally, another research has demonstrated that administration of butyrate-producing Roseburia could inhibit colon tumorigenesis induced by a high-fat diet (Chen et al., 2020). However, the precise anti-tumorigenic effects of microbiome-derived butyrate in conjunction with 5-FU within the context of CRC, along with the underlying intricate mechanisms, remain shrouded in ambiguity. Unraveling the association between novel bacteria implicated in CRC resistance and the molecular mechanisms involved may pave the way for the development of innovative diagnostic and therapeutic approaches, ultimately enhancing survival outcomes for CRC patients.

In the current study, we investigated microbiome composition in CRC patients with short or long resistance-free survival (RFS) by 16S rRNA sequencing. Our findings revealed a diminished intratumor microbiome diversity in patients with short RFS, coupled with an enrichment of butyrate-producing bacteria in this cohort. The intratumor microbiome signature, in conjunction with pre-operative circulating microbiome DNA (cmDNA), demonstrated a high predictive accuracy for CRC resistance. We found that Roseburia, a prominent butyrate-producing bacterium, might serve as a potential promoter of CRC resistance. Additionally, butyrate supplementation could directly enhance drug resistance through modulating PI3K/AKT pathway. Findings position butyrate as a potential anti-tumor agent and a valuable adjunct to chemotherapy in the treatment of CRC.

## 2 Materials and methods

### 2.1 Patients and samples

This study was approved by the ethics committee of The Second Affiliated Hospital of Xi’an Jiaotong University. Patents and related samples were selected from specimen repository in our center between 2013 and 2023 according to follow criteria: 1. CRC patients; 2. Received radical surgery; 3. Resistance or sensitive in 3 years. We excluded subjects with a prior history of cancer and antibiotic use (less than 1 month) or neoadjuvant therapy before surgery. Tumor response was assessed and categorized as a complete response (CR), partial response (PR), stable disease (SD), or progressive disease (PD). Patients with CR and PR were defined as resistant group (R) and those with SD and PD were defined as non-resistant group (NR). After matching for various clinicopathologic variables, 19 patients were included as R group, with 12 patients chosen to NR group (16S cohort). Detailed clinical and pathologic information on the patients is presented in Table 1. Tumor and normal specimens were frozen in a liquid nitrogen tank immediately after resection in a sterile environment, and then transferred to −80°C until processing for DNA extraction (Yu et al., 2023). Another cohort of 28 treatment-naive CRC patients with R (18) and NR (10) were enrolled in this study according to above-mentioned criteria (cmDNA cohort). Detailed clinical and pathologic information on the patients is presented in Table 2. Samples were collected in a sterile environment maintained at a temperature of 4°C to preserve the integrity of the biological materials. All samples were processed under strict sterile conditions with specific attention to temperature control. Tumor and normal specimens were immediately frozen in a liquid nitrogen tank at −196°C post-resection and stored at −80°C until further DNA extraction (Yu et al., 2023). Plasma samples were collected in pre-chilled EDTA tubes and centrifuged at 4°C to separate plasma from cellular components, followed by a second centrifugation at 16,000 g to remove any remaining cellular debris (Maurer et al., 2024). The plasma was then stored at −80°C until DNA extraction.

**TABLE 1:** Clinical characteristics of patients in 16S cohort.

Clinical characteristics	R (n = 19)	NR (n = 12)	*p*-value
Resistance-free survival (years), mean ± SD	0.81 ± 0.45	4.03 ± 0.89	<0.05
Age (years), mean ± SD	56.83 ± 7.36	55.98 ± 8.17	>0.05
Gender			>0.05
Female	8	7	
Male	11	5	
BMI (kg/m2), mean ± SD	25.11 ± 2.78	24.89 ± 3.02	>0.05
Tumor diameters (cm), mean ± SD	1.94 ± 0.67	1.45 ± 0.36	<0.05
TNM stage			<0.05
Ⅰ	4	12	
Ⅱ	6	0	
Ⅲ	9	0	
Pathology			<0.05
Adenocarcinoma	17	9	
Squamous cell carcinoma	2	3	
Others	1	0	

R, resistance group; NR, non-resistance group; BMI, body mass index.

**TABLE 2:** Clinical characteristics of patients in validation set.

Clinical characteristics	R (n = 18)	NR (n = 10)	*p*-value
Resistance-free survival (years), mean ± SD	0.89 ± 0.63	3.91 ± 1.12	<0.05
Age (years), mean ± SD	57.04 ± 6.73	55.76 ± 7.34	>0.05
Gender			>0.05
Female	9	6	
Male	9	4	
BMI (kg/m2), mean ± SD	24.83 ± 3.84	24.37 ± 2.61	>0.05
Tumor diameters (cm), mean ± SD	2.08 ± 0.79	1.31 ± 0.56	<0.05
TNM stage			<0.05
Ⅰ	3	10	
Ⅱ	5	0	
Ⅲ	10	0	
Pathology			<0.05
Adenocarcinoma	16	7	
Squamous cell carcinoma	1	3	
Others	1	0	

R, resistance group; NR, non-resistance group; BMI, body mass index.

We applied transparent exclusion criteria to ensure the study’s rigor. Patients with a history of prior cancer, recent antibiotic use within the last month, or those who underwent neoadjuvant therapy were excluded to minimize confounding factors that could affect treatment response and microbiome composition. To address potential biases in selecting resistant and non-resistant patient groups, we meticulously matched patients based on clinicopathologic variables. This approach aimed to ensure that any observed differences in resistance were not due to confounding variables but rather due to the biological differences between the groups. The rationale for assigning patients to resistant and non-resistant groups was based on their tumor response to treatment. This classification allowed us to investigate the differences in microbiome composition and its association with treatment outcomes.

### 2.2 DNA extraction and 16s gene sequencing

16S rRNA sequencing was performed by the Microbial Genome Research Center (IMCAS, Beijing, China). DNA extraction was performed using the QIAamp DNA Mini Kit (Qiagen) following the manufacturer’s protocol with modifications to accommodate tissue samples. The extraction process included initial incubation at 56°C for 10 min, followed by sequential washes and elution steps, all conducted at 4°C to preserve DNA integrity (Maurer et al., 2024). The V3 and V4 regions of the bacterial 16S rDNA gene were amplified using primers designed to bind at specific annealing temperatures, optimized for our samples (Yu et al., 2023). Purified amplicons were pooled in equimolar amounts and paired-end sequenced (2 × 250) on an Illumina MiSeq platform according to standard protocols. FLASH software (version 1.2.11, https://ccb.jhu.edu/software/FLASH/index.shtml) was used to merge paired-end reads from next-generation sequencing. Low-quality reads were filtered by FASTX Toolkit (version 1.2.11, http://hannonlab.cshl.edu/fastx_toolkit/), and chimera reads were removed by USEARCH (version 11) program’s UCHIME command and the “GOLD” database. After a random selection of 20,000 reads, the taxonomical classification of reads was determined using the RDP classifier (version 2.7) to generate the composition matrices at the level of the phylum to the genus. A bootstrap value > 0.8 was considered as high-confidence taxonomy assignment, while low-confidence sequences were labeled as unclassified assignment. Alpha diversity in our samples were calculated and displayed by vegan R package. Principal coordinate analysis (PCoA) was performed to visualize the Beta diversity between different groups. The linear discriminant analysis (LDA) effect size (LEfSe) method was used to detect microbial biomarkers (|LDA| score >2.5 and *p* < 0.05) among different groups. Lasso regression model was used to further selection of microbial biomarkers. The genus predicting score was generated as follows: genus predicting score = β1x1 + β2x2 + + βixi where βi is the coefficient of each genus and xi is the relative abundance of each genus.

### 2.3 Circulating microbiome DNA sequencing and analysis

Whole blood was collected in EDTA tubes after skin surfaces were sterilized twice and processed immediately to minimize contamination. Plasma and cellular components were separated by centrifugation at 1600 *g* for 10 min at 4°C. To further reduce the risk of contamination, all centrifugation steps were performed in a certified DNA-free environment. Plasma was centrifuged a second time at 16,000 g at 4°C to remove any remaining cellular debris and stored at −80°C until the time of DNA extraction (Yu et al., 2023). NGS cfDNA libraries were prepared for whole genome sequencing using 10–250 ng of cfDNA. Briefly, the Qubit dsDNA HS Assay Kit was used to measure cfDNA concentrations according to the manufacturer’s recommendations. To ensure the quality and purity of the extracted DNA, we performed spectrophotometry and electrophoresis before and after the extraction process. Then, genomic libraries were prepared using the VAHTS Universal DNA Library Prep Kit for Illumina V3 (Maurer et al., 2024). Whole genome libraries were sequenced using 100-bp paired-end runs on the DNBSEQ-T7, which was performed by Geneplus-Beijing Institute (Beijing, China). All sequence reads were first mapped to reference sequence hg19 (Human Genome version 19) using Bowtie2 (v2.3.5.1) with default parameters. Reads that mapped to human genome were removed using Samtools software. The filtered reads were mapped to NCBI microbial reference genome databases using k-mer-based algorithm with Kraken. Relative abundance at bacterial genus level were estimated by Braken with recommended parameters. We used the MaAslin2 software to get genera with top predictive ability in discovery set with q value <0.25. Random forest model with selected genera as input was constructed with the caret package and the randomForest R package. The receiver operating characteristics (ROC) curve and class predictions were generated by pROC R package. Sequencing was conducted on the DNBSEQ-T7 platform with a minimum of 30x coverage per sample to ensure high-quality data output. The sequencing run parameters were optimized for read length and quality, with an average Q-score of 30, ensuring accurate base calling and minimal errors.

### 2.4 Cell culture

The human colorectal cancer cell line HCT15 and HCT8 was purchased from Shanghai Institute of Biochemical Cell Science, Chinese Academy of Sciences. In brief, cells were cultured in high sugar complete medium (DMEM) (Gibco, 11,995,065, United States) supplemented with 10% fetal bovine serum (FBS) (Sciencell, 0500, United States) and 1% penicillin–streptomycin (HyClone, SV30010, United States). Medium was changed every 2 days and passaged when reaching 80% confluence. All experiments were performed with mycoplasma-free cells. For groups supplemented with cholesterol, different concentrations were added into DMEM. 5-FU was also added to evaluate drug resistance. Cells were cultured in a CO2 incubator at 37°C with 5% CO2 and 95% humidity. The culture medium was refreshed every 48 h to maintain optimal growth conditions, and cells were passaged upon reaching 80%–90% confluence to ensure healthy cell growth and minimize contact inhibition.

### 2.5 Quantitative real-time PCR (qPCR)

Total RNA was extracted with TRIzol (R401, Vazyme) and subsequently reverse transcribed into cDNA with HiScript II Q RT SuperMix (R222-01, Vazyme) in preparation for qPCR. qPCR was consequently conducted with an Applied Biosystems 7,500 device with ChamQ SYBR qPCR Master Mix (Q331-02, Vazyme). 2^−ΔΔCT^ approach was employed to calculate the relative expression levels, in turn normalized to β-actin. The sequences of all primers are illustrated in Table 1 qPCR reactions were performed in a final volume of 20 μL, containing 10 μL of ChamQ SYBR qPCR Master Mix, 2 μL of cDNA, and 0.4 μM of each primer. The cycling conditions included an initial denaturation at 95°C for 10 min, followed by 40 cycles of 95°C for 15 s and 60°C for 1 min. Melt curve analysis was performed to confirm the specificity of the amplification.

### 2.6 Cell proliferation and migration

Cells were seeded into a 96-well plate and incubated in culture medium with variable concentrations of butyrate for 24 h and 48 h. Subsequently, the cells were labeled using a Cell Counting Kit-8 (CCK8) (Biosharp, BS350A) for 2 h. The absorbance of each well was measured with a microplate reader set at 450 nm. Wound healing assays were performed to evaluate the migration capabilities. HCT15 and HCT8 cells were grown in 6-well plastic dishes and treated with 100 mM butyrate or control for 24 h. Quantitive analysis was performed through ImageJ software. Each experiment was performed in triplicate. Butyrate was used at concentrations of 0, 1, 5, and 10 mM to assess its dose-dependent effects on cell proliferation. Cells were incubated with butyrate for 24 and 48 h in a humidified incubator at 37°C and 5% CO2. The absorbance was measured at 450 nm using a microplate reader to quantify cell proliferation.

### 2.7 Quantitative and statistical analysis

To validate the predictive accuracy of our Lasso regression model, we utilized cross-validation techniques. Specifically, we employed k-fold cross-validation, where the data is divided into k subsets, and the model is trained and tested k times, each time using a different subset as the test data. This process allows for a more robust assessment of the model’s performance. We also calculated the mean squared error (MSE) to quantify the average squared difference between the predicted and actual values, providing a measure of the model’s accuracy. Additionally, we generated ROC curves to evaluate the model’s ability to discriminate between patients with different RFS outcomes.

In our survival analysis, we included additional clinical and pathological variables that may potentially act as confounders. To balance the distribution of confounding variables between comparison groups and reduce selection bias, we employed propensity score matching. This technique involves calculating a propensity score for each patient based on the covariates and then matching patients in the resistant and non-resistant groups. This approach allowed us to compare survival outcomes more robustly. To assess the robustness of our findings to different model specifications and to evaluate the impact of potential confounding variables on our results, we conducted sensitivity analyses. These analyses included testing various models with different combinations of covariates to ensure that our results were consistent and reliable.

Data were expressed as the mean ± standard deviation (SD). To determine the statistical significance of observed differences, we utilized one-way or two-way analysis of variance (ANOVA), followed by Tukey’s *post hoc* test for multiple comparisons when appropriate. For direct comparisons between two groups, we employed unpaired two-tailed Student's t-tests. The levels of statistical significance were set at *p < 0.05, **p < 0.01, ***p < 0.001, and ****p < 0.0001. All calculations were performed using Prism version 9.0 (GraphPad Software, Inc.) on a Windows 11 operating system. Data were analyzed using one-way or two-way ANOVA with Tukey’s *post hoc* test for multiple comparisons. For direct comparisons, unpaired two-tailed Student's t-tests were employed. The significance level was set at *p* < 0.05, with adjustments for multiple comparisons. We revisited our statistical analyses to ensure that we have appropriately accounted for any potential biases in patient selection. This included the use of appropriate statistical methods to control for confounding variables and to assess the impact of potential biases on our results.

## 3 Results

### 3.1 Tumor microbial diversity is associated with resistance in patients with CRC

To investigate the relationship between colorectal microbiome composition and chemoresistance in CRC, we established a well-characterized cohort. This cohort included patients experiencing post-surgery drug resistance (resistance [R] group, median RFS 0.81 years) and long-term survivors without resistance for over 3 years (non-resistance [NR] group, median RFS 4.03 years). Both groups were matched for age, gender, BMI, clinical stage, tumor dimensions, and pathological features, as shown in Table 1. Notably, the R group exhibited a more advanced TNM stage, consistent with known tumor resistance characteristics. Bacterial DNA was extracted from 31 patients, including paired CRC tumor and adjacent normal tissue samples (12 R and 19 NR). We conducted taxonomic profiling using 16S rRNA gene sequencing to assess microbial composition and its potential association with CRC resistance.

**FIGURE 1:**
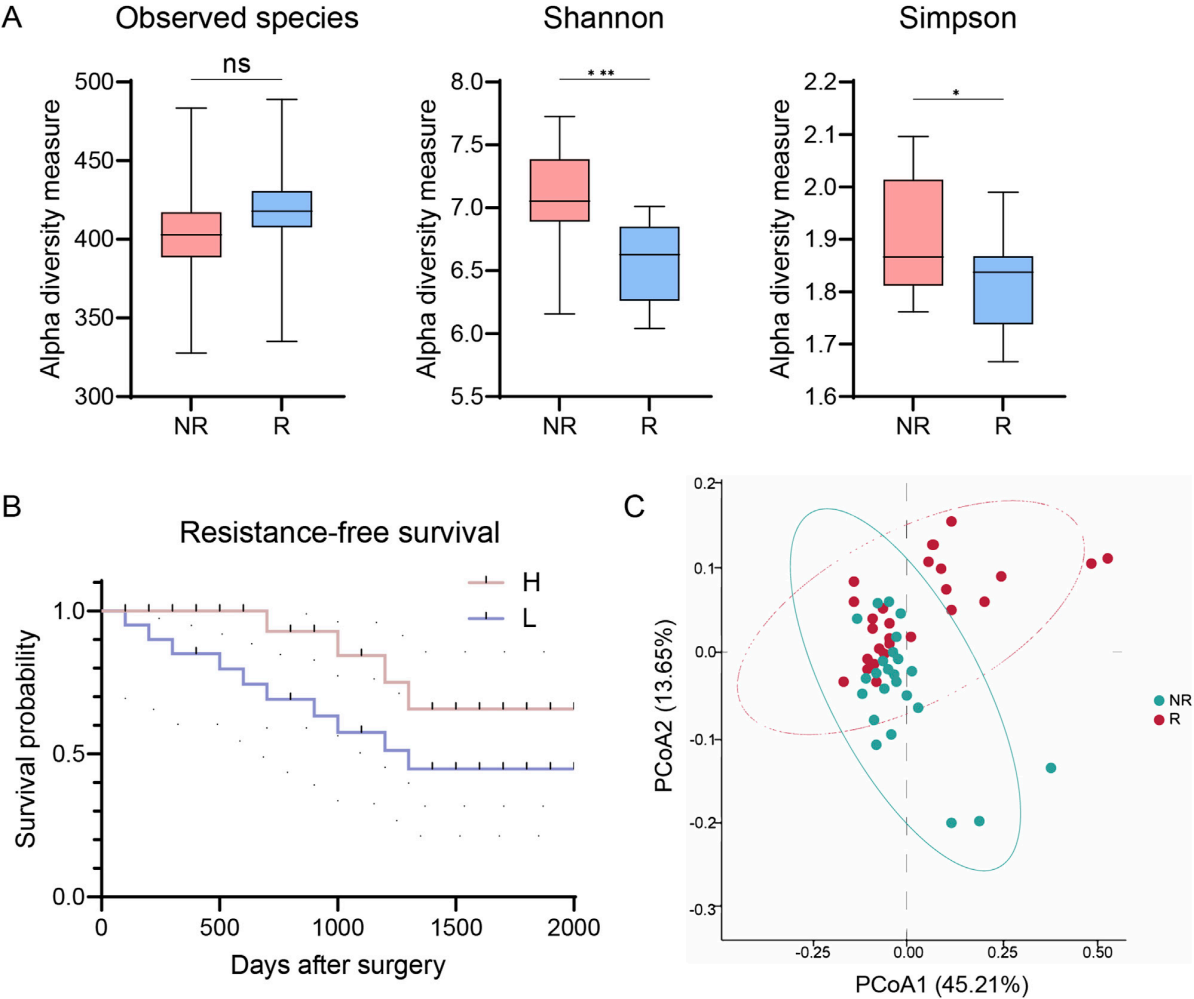
Intratumor microbial diversity correlates with resistance of patients with CRC. **(A)** Alpha diversity in R and NR groups (observed species, Shannon, and Simpson indices). **(B)** Kaplan-Meier plot of patients with CRC defined by alpha diversity. **(C)** PCoA using Bray-Curtis metric distances of beta diversity. R, resistant group; NR, non-resistant group; H, high diversity; L, low diversity; PCoA, principal coordinate analysis. The error bars indicate the standard deviations.

Our initial analysis quantified microbial diversity within tumor samples using metrics such as observed taxonomic units, Shannon, and Simpson indices. We found that the alpha diversity of the tumor microbiome, reflecting both abundance and diversity of microbial species, was significantly higher in NR patients compared to R patients (*p* < 0.001 for Shannon and *p* < 0.05 for Simpson, Figure 1A). Stratifying the cohort based on the median Shannon index diversity score, we observed that patients with low alpha diversity had decreased RFS compared to those with high diversity (HR = 2.42, 95% CI: 0.8352–7.011, *p* = 0.0494, Figure 1B).

To further understand the role of microbiome diversity in chemoresistance, we compared overall microbiome composition between R and NR groups using microbial beta diversity. Principal coordinate analysis (PCoA) with Bray-Curtis distance measurements revealed distinct clustering patterns (Figure 1C). Analysis of Similarity (ANOSIM) confirmed significant differences in microbiome composition between the groups (R = 0.218, *p* < 0.05, Figure 1C). These results highlighting the intricate relationship between the tumor microbiome diversity and therapeutic resistance in CRC.

### 3.2 Tumor microbiome communities are remarkedly different between R and NR patients

**FIGURE 2:**
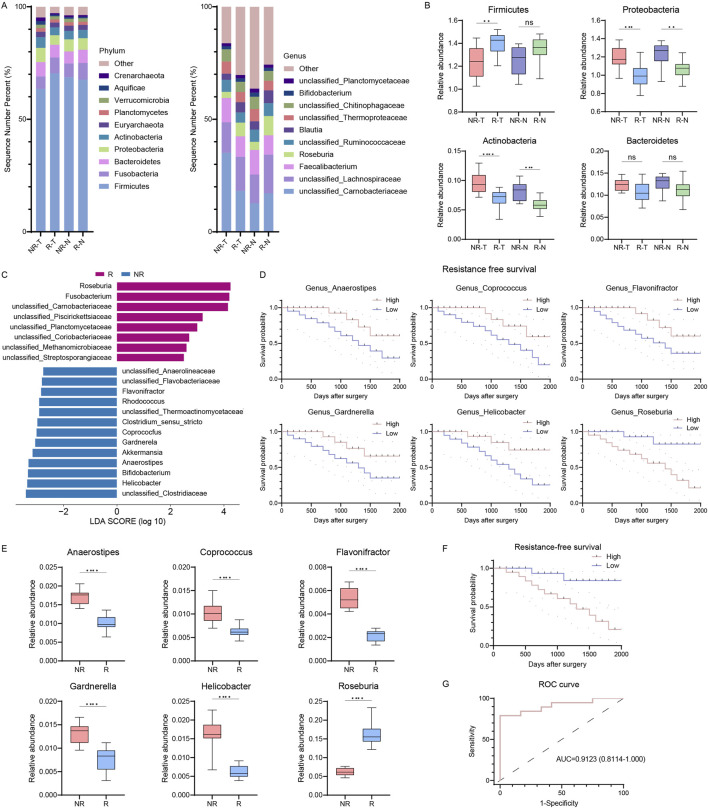
Intratumor microbiome communities are significantly different between R and NR patients. **(A)** Bar plots of the phylum (left) and genus (right) taxonomic levels in R and NR patients with CRC. Relative abundance is used. **(B)** Phylum differences between R and NR patients. **(C)** LDA score of features with different abundances between R and NR groups. The criteria for differential feature is an LDA score >2.5. **(D)** Kaplan-Meier estimates for RFS probability of patients with different abundances of intratumor microbes. Up, Anaerostipes, Coprococcus, and Flavonifractor; right, Gardnerella, *Helicobacter*, and Roseburia. **(E)** Six differentially abundant genera in genus predicting score. **(F)** Kaplan-Meier plot of patients with CRC defined by genus predicting score. **(G)** ROC analysis of genus predicting score as predictive of RFS. R, resistant group; NR, non-resistant group; T, tumor tissues; N, normal colorectal tissues; LDA, linear discriminant analysis; RFS, resistance-free survival; ROC, receiver operating characteristics. The error bars indicate the standard deviations.

Building on the correlation between microbial diversity and RFS in CRC patients, we examined microbial community differences between R and NR patients. At the phylum level, Firmicutes, Bacteroidetes, Proteobacteria, and Actinobacteria were predominant in both groups, regardless of tissue type (Figure 2A). Notably, R patients showed increased Firmicutes and Fusobacteria and decreased Proteobacteria and Actinobacteria compared to NR patients (Figure 2B). At the genus level, Faecalibacterium, Roseburia, Blautia, and Bifidobacterium were abundant in both groups (Figure 2A). However, Roseburia increased, while Faecalibacterium and Bifidobacterium decreased in the R group (Figure 2B).

To identify differential microbial signatures, we conducted a linear discriminant analysis of effect size (LEfSe) at the genus level, revealing 22 features distinguishing R from NR groups (Figure 2C). Roseburia and Fusobacteria were prominent biomarkers for the R group, while Bifidobacterium, *Helicobacter*, and Akkermansia were biomarkers for the NR group. Using a Lasso regression model, we identified six genera (Roseburia, *Helicobacter*, Gardnerella, Flavonifractor, Coprococcus, and Anaerostipes) as potential biomarkers to differentiate between groups, significantly correlating with RFS (Figure 2D). The relative abundances of these genera differed significantly between R and NR groups (Figure 2E). Patients were classified into high- and low-risk groups based on a median predicting score from these genera. The Kaplan-Meier survival curve showed significantly shorter RFS in the high-risk group (HR = 4.202, 95% CI: 1.470–12.01, *p* = 0.0074, Figure 2F). The genus predicting score remained an independent RFS predictor in multivariate Cox regression, with an AUC of 0.9123, indicating high predictive accuracy (Figure 2G). These results indicate significant differences in tumor microbiome communities between R and NR patients.

### 3.3 cmDNA signatures as biomarkers for CRC resistance

**FIGURE 3:**
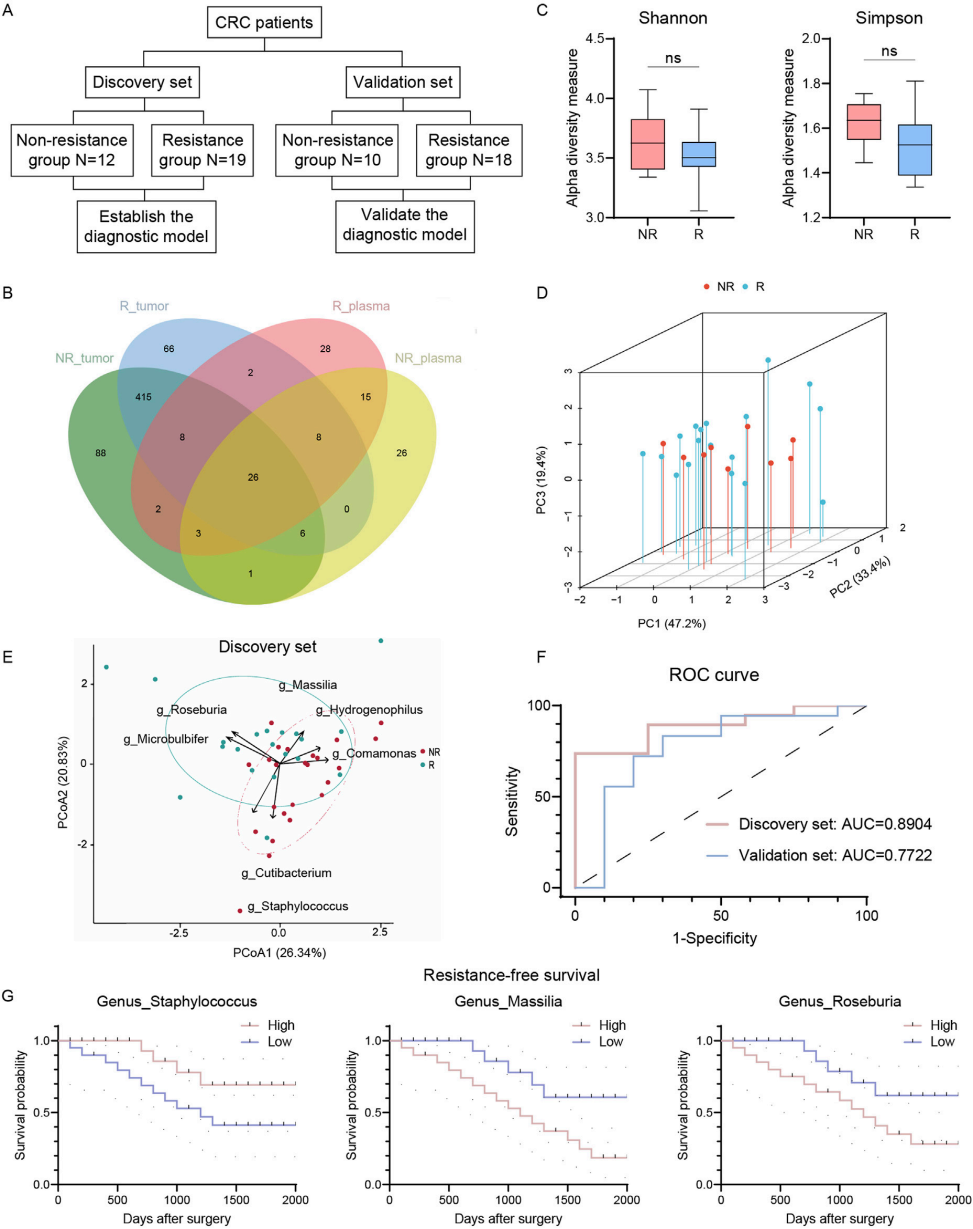
Circulating microbiome DNA could distinguish R and NR patients. **(A)** Flowchart of circulating microbiome DNA analysis. **(B)** Venn plot of shared genus in tumor and plasma. **(C)** Alpha diversity of circulating microbiome DNA in R and NR patients (Shannon and Simpson indices). **(D)** PCoA of circulating microbiome DNA in R and NR patients using Bray-Curtis metric distances of beta diversity. **(E)** PCA using circulating microbiome DNA biomarkers in discovery set. **(F)** ROC analysis of circulating microbiome DNA signature as predictive of R patients in discovery and validation sets. **(G)** Kaplan-Meier estimates for RFS probability based on the abundance levels of microbes in plasma. Left, *Staphylococcus*; middle, Massilia; right, Roseburia. R, resistant group; NR, non-resistant group; PCoA, Principal coordinate analysis; PCA, principal-component analysis, ROC, receiver operating characteristics. The error bars indicate the standard deviations.

Recognizing cmDNA’s potential as a biomarker in cancer diagnostics, we expanded our study to explore its association with CRC resistance (Figure 3A, cmDNA cohort). We recruited 28 CRC patients and analyzed their plasma samples alongside previously collected ones using whole-genome sequencing. Demographic and clinical characteristics were comparable between R and NR groups (Table 2). We identified 26 shared genera in both plasma and tumor tissues, including Roseburia and Fusobacteria, previously identified as tumor tissue biomarkers (Figure 3B). Consistent with tumor tissue observations, NR patients had higher cmDNA alpha diversity, though not statistically significant (*p* = 0.2038 for Shannon and *p* = 0.0980 for Simpson, Figure 3C). PCoA using Bray-Curtis distance metrics showed discernible differences in cmDNA profiles between R and NR groups (ANOSIM R = 0.086, *p* < 0.05, Figure 3D), suggesting cmDNA’s potential role in reflecting CRC resistance-associated microbial landscapes.

Patients were divided into discovery and validation sets for model calibration and validation (Figure 3A). Using the MaAslin2 algorithm, we identified seven genera with predictive probability. Roseburia, Massilia, and Microbulbifer were enriched in the R group, while Cutibacterium, Comamonas, *Staphylococcus*, and Hydrogenophilus were enriched in the NR group. Principal-component analysis revealed distinct clustering patterns between R and NR groups, highlighting divergent microbial signatures associated with chemoresistance (Figure 3E). A random forest model based on these seven genera achieved an AUC of 0.8904 in the discovery set, indicating high discriminatory capacity (Figure 3F). Validation set analysis yielded an AUC of 0.7722, maintaining acceptable predictive accuracy. *Staphylococcus*, Massilia, and Roseburia were significantly correlated with RFS (Figure 3G). These findings suggest cmDNA signatures as promising non-invasive biomarkers for preoperative chemoresistance prediction in CRC.

### 3.4 Microbiota-derived butyrate supplementation promotes CRC resistance

**FIGURE 4:**
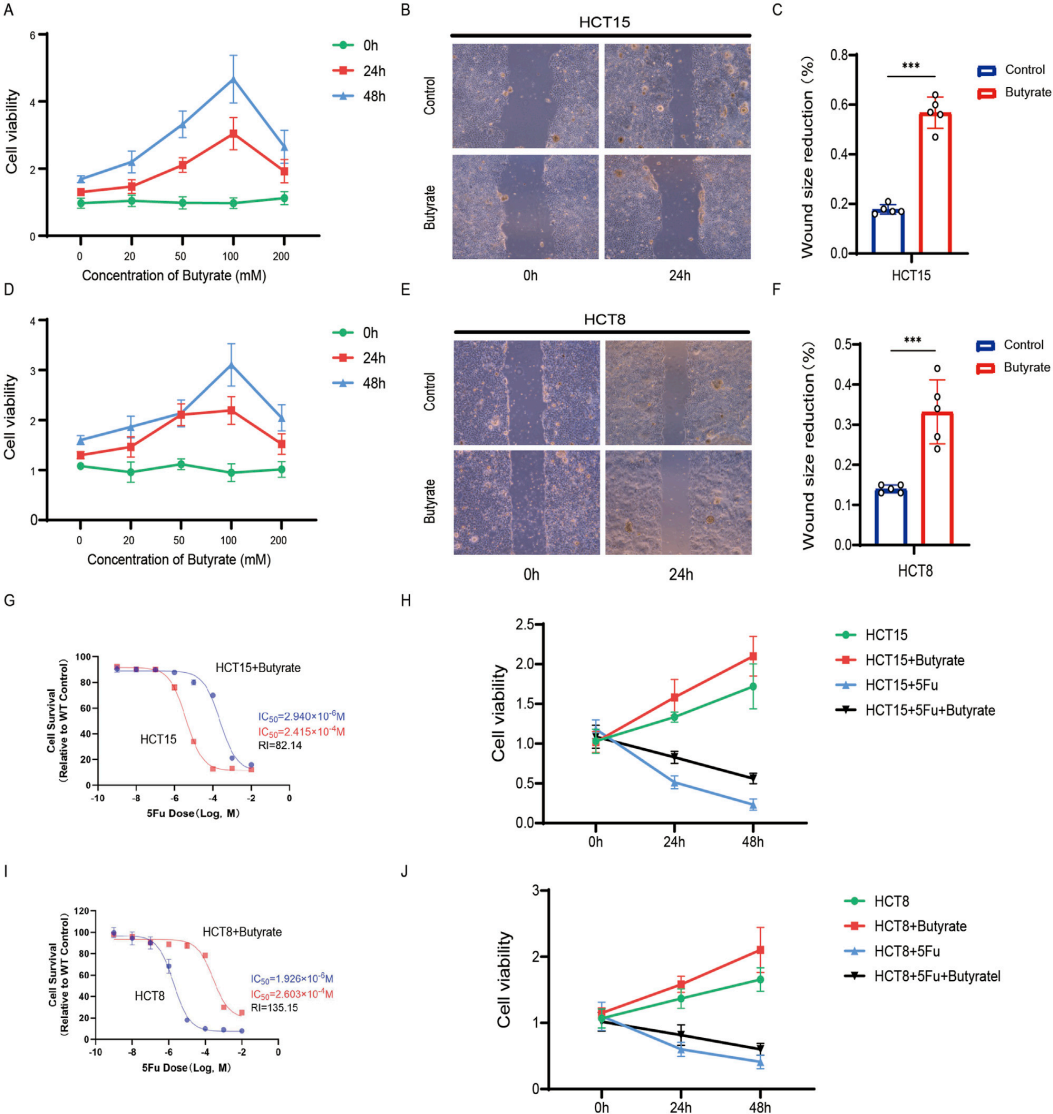
Microbiota-derived butyrate supplementation promotes CRC resistance. **(A)** CCK8 assay of HCT15 treated with butyrate at different time and concentrations. Data depict one representative experiment of five independent experiments; duplicate conditions for each experiment. **(B)** Wound-healing assay of HCT15 treated with butyrate (100 mM). Data depict one representative experiment of five independent experiments; duplicate conditions for each experiment. **(C)** Quantitative analysis of aforesaid wound-healing assay. **(D)** CCK8 assay of HCT8. **(E)** Wound-healing assay of HCT8. **(F)** Quantitative analysis. **(G)** Cell counting assay under different doses of 5-FU for 24 h with or without butyrate treatment of HCT15 (100 mM). The IC50 values in these cells were further calculated with Graphpad Prism 7.0. **(H)** CCK8 assay of HCT15 treated with or without butyrate and 5-FU after 24 h or 48 h. **(I)** Cell counting assay of HCT8. **(J)** CCK8 assay of HCT8. *p* values were calculated by non-paired Student’s tests. ∗*p* < 0.05, ∗∗*p* < 0.01, and ∗∗∗*p* < 0.001. The error bars indicate the standard deviations.

Our 16S rRNA sequencing analysis revealed a reduction in microbial diversity within tumor samples of patients with resistance, with an enrichment of butyrate-producing bacteria in this cohort, particularly Roseburia, as being significantly associated with chemoresistance. This prompted us to investigate butyrate as a potential mediator of chemoresistance. Given its production by bacteria identified as biomarkers for resistance, we hypothesized that butyrate might be a key factor in promoting chemoresistance in CRC. We examined the impact of butyrate supplementation on CRC cell line proliferation. The CCK8 assay showed that butyrate enhances HCT15 cell proliferation in a time- and concentration-dependent manner (Figure 4A). A concentration of 100 mM, demonstrating the most pronounced effect, was selected for further experiments. We then assessed butyrate’s influence on CRC cell migration. The wound-healing assay indicated that butyrate significantly enhanced HCT15 cell migration (Figure 4B), with quantitative analysis supporting these findings (Figure 4C). Similar results were observed in HCT8 cells (Figures 4D–F). To assess butyrate’s effect on chemoresistance, 5-FU was supplemented at varying concentrations. Cell survival analysis showed that butyrate treatment increased the IC50 value (2.940*10^−6^ M vs. 2.415*10^−4^ M, RI = 82.14), indicating increased drug resistance in HCT15 cells (Figure 4G). Increased cell viability was observed under butyrate supplementation, regardless of 5-FU administration, after 24 and 48 h (Figure 4H). Similar phenomena were noted in HCT8 cells (Figures 4I,J). These results suggest that butyrate modulates CRC cell behavior, enhancing proliferation, migration, and chemoresistance.

### 3.5 Butyrate-induced activation of the PI3K/AKT pathway

**FIGURE 5:**
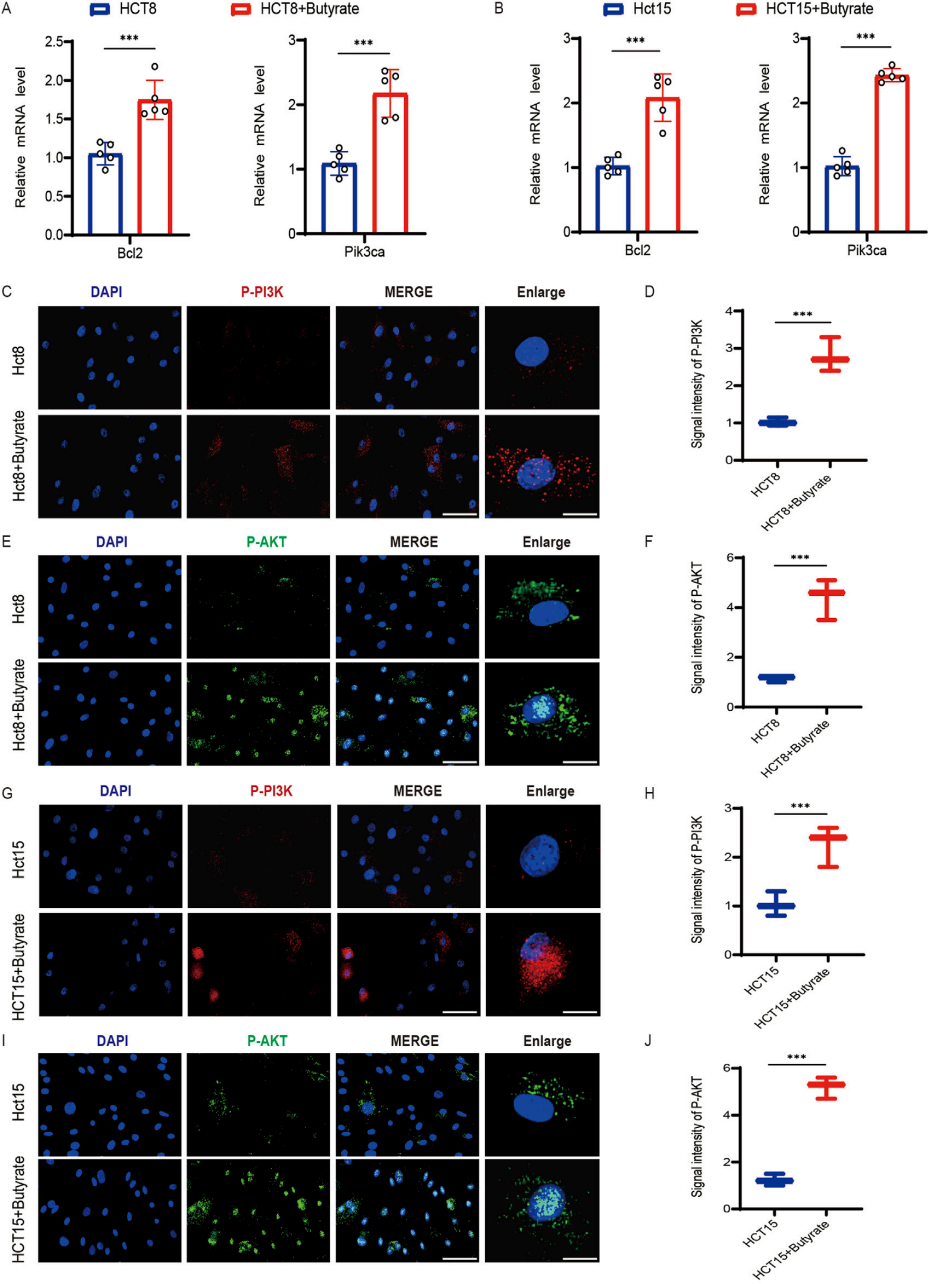
Effects of butyrate on the activation of the PI3K/AKT pathway in CRC cells. **(A)** qPCR analysis of Bcl2 and Pik3ca mRNA levels in HCT8 cells following butyrate treatment. **(B)** qPCR analysis in HCT15 cells following butyrate treatment. **(C)** Immunofluorescence analysis of phosphorylation of PI3K (P-PI3K) in HCT8 cells exposed to butyrate (scale bar, 40μm, enlarged, 12 μm). **(D)** Quantitative measurements of signal intensity. **(E)** Immunofluorescence analysis of phosphorylation of AKT (P-AKT) in HCT8 cells exposed to butyrate (scale bar, 40μm, enlarged, 12 μm). **(F)** Quantitative measurements of signal intensity. **(G)** Immunofluorescence analysis of P-PI3K in HCT15 cells (scale bar, 40μm, enlarged, 12 μm). **(H)** Quantitative measurements of P-PI3K. **(I)** Immunofluorescence analysis of P-AKT (scale bar, 40μm, enlarged, 12 μm). **(J)** Quantitative measurements. *p* values were calculated by non-paired Student’s tests. ∗*p* < 0.05, ∗∗*p* < 0.01, and ∗∗∗*p* < 0.001. The error bars indicate the standard deviations.

To explore the molecular mechanisms underlying butyrate’s effects on chemoresistance, we conducted a thorough examination of the expression levels of Bcl2, a key modulator of apoptosis, as well as the activation status of PI3K and AKT, integral to cell survival and proliferation. qPCR analysis revealed a significant upregulation of Bcl2 mRNA in butyrate-treated HCT8 cells, indicative of its anti-apoptotic influence (Figure 5A). Moreover, butyrate supplementation led to a notable increase in Pik3ca expression, pointing towards a potential intensification of PI3K/AKT signaling activity (Figure 5A). Similar phenomena were observed in the gene expression profiles of butyrate-treated HCT15 cells (Figure 5B). Further immunofluorescence analysis demonstrated an enhancement in the levels of phosphorylated AKT (P-AKT) and phosphorylated PI3K (P-PI3K) in butyrate-exposed HCT8 cells (Figures 5C,E), with quantitative measurements of signal intensity corroborating this activation (Figures 5D,F). Activation of the PI3K/AKT pathway was also discernible in the immunofluorescence analysis of butyrate-treated HCT15 cells (Figures 5G–J). These findings collectively suggest that butyrate induced the PI3K/AKT pathway activation to increase the drug resistance.

## 4 Discussion

CRC represents the preeminent malignant neoplasm of the gastrointestinal tract, with a global incidence and mortality rate that persist in their upward trajectory (Li et al., 2024c; Guo et al., 2022a). CRC cells have been observed to exhibit a capacity to develop an augment resistance to conventional chemotherapeutic agents, such as 5-FU. It is exacerbated by the gut microbiota, which plays a pivotal role in both resistance and cancer progression (Li et al., 2024c; Guo et al., 2023). Numerous commensal bacterial species have been correlated with the advancement of CRC and are increasingly recognized as potential diagnostic markers (Zhang et al., 2023; Wu et al., 2023). In this study, we have delineated a correlation between the diversity and composition of the tumor microbiome and the resistance to CRC treatment. Our findings underscore that a diminished diversity of the tumor microbiome is significantly associated with a decreased RFS. Moreover, cmDNA signatures have exhibited notable predictive capabilities in both our discovery and validation cohorts. An additional salient finding was the perturbation of butyrate-producing bacteria, such as Roseburia, which correlated with a diminished RFS. Intriguingly, our experiments have also revealed that butyrate supplementation can actively promote chemoresistance in CRC.

Accumulating evidence indicates that commensal bacteria play an indispensable role in the immune system and tumor progression (Zhang et al., 2023; Yu et al., 2022). However, the precise relationship between the tumor microbiome and resistance to CRC remains unevenly elucidated. There is a growing consensus that decreased tumor microbiome diversity is associated with poorer survival outcomes among cancer patients (Tito et al., 2024; Chen et al., 2022b). Overall, we have performed a comprehensive analysis of the intratumor microbiome within a cohort of CRC patients, categorized as either R or NR groups. Our findings suggested that CRC tumor microbiome diversity was significantly diminished in the R group and was associated with reduced RFS, implying that the tumor microbiome may exert an influence on tumor resistance. Importantly, we identified a signature comprising six tumor bacterial genera (Roseburia, *Helicobacter*, Gardnerella, Flavonifractor, Coprococcus, and Anaerostipes), which may serve as potential biomarkers for stratifying patients based on resistance. Additionally, *Fusobacterium* was found to be enriched in resistant tumor tissues, a finding consistent with prior research highlighting its critical association with CRC progression and drug resistance (Bullman et al., 2017). This genus may contribute to tumor cell survival and proliferation through its distinctive metabolic activities, modulation of the immune response, or interactions with other microorganisms, while simultaneously diminishing tumor cell sensitivity to chemotherapeutic agents (Tito et al., 2024). Furthermore, genus predicting scores based on specific bacterial genera in tumor or normal colorectal tissues demonstrated robust predictive accuracy for RFS. Building on the pioneering work of Poore et al., who proposed a novel cancer diagnostic approach with high accuracy through microbiome analyses of blood (Matsushita et al., 2021), we discovered that cmDNA signatures exhibited promising predictive performance for resistance in both our discovery and validation cohorts. CmDNA holds the potential to evolve into a non-invasive biomarker for resistance prediction in CRC. However, large-scale studies are warranted to further substantiate the reliability of tumor or cmDNA signatures in cancer diagnostics.

As identified before, butyrate-producing bacteria was enriched, particularly Roseburia, as being significantly associated with chemoresistance. Roseburia, a prominent butyrate-producing bacterium, has been recognized for its capacity to mitigate inflammation within the intestinal tract (Shen et al., 2018; Gu et al., 2024). In our study, Roseburia was observed to be enriched in both tumor and adjacent normal tissues of the R group and was correlated with a reduced RFS. This observation is congruent with the findings of Peters et al., who reported an association between Roseburia and diminished survival rates in patients with lung cancer (Peters et al., 2019). Previous research has highlighted the multifaceted roles of butyrate, including its anti-inflammatory, antioxidant, and tumor-suppressive effects within the intestinal milieu (Ma et al., 2024), which has undergone evaluation in clinical trials as a potential anticancer therapeutic for the treatment of human malignancies (Li et al., 2024c). It is noted for its ability to inhibit cell proliferation at higher concentrations while paradoxically promoting cell proliferation at lower concentrations (Matsushita et al., 2021), which has been demonstrated to enhance tumor cell proliferation in prostate cancer (Matsushita et al., 2021). Despite butyrate’s established capability to inhibit proliferation and induce apoptosis, there exists a paucity of comprehensive data elucidating its regulatory influence on CRC resistance. Our findings revealed a disturbance in various butyrate-producing bacteria, such as Roseburia, within the tumor and normal tissues of CRC patients. Functional assays further indicated that butyrate supplementation could enhance drug resistance and promote tumor progression. Future research endeavors should unravel the precise mechanisms by which butyrate-producing bacteria contribute to CRC drug resistance. There is also a need to explore strategies to optimize CRC treatment outcomes by modulating the gut microbiota, potentially through targeted interventions that harness the metabolic byproducts of these bacteria.

Our study reveals that butyrate, originating from the microbiome, significantly stimulates the PI3K/AKT signaling pathway, which appears to underpin the chemoresistance observed in CRC cells. This pathway has been established as a key mediator of drug resistance in various cancer types, including non-small cell lung cancer (NSCLC) (Shi et al., 2022) and gastric cancer (Ren et al., 2023). The resistance to chemotherapeutic agents is attributed to a complex array of mechanisms, such as the upregulation of oncogenes and growth factors like VEGF, c-myc, and cyclin D1 (Lu et al., 2020). Consistent with our observations, the activation of the PI3K/AKT pathway may also lead to the upregulation of anti-apoptotic proteins, including Bcl-2 (Chen et al., 2022a). Additionally, the activation of downstream signaling molecules like mTOR can promote cell proliferation and inhibit cell death, along with the regulation in epithelial-mesenchymal transition (EMT) (Wang et al., 2021; Guo et al., 2024). Future research endeavors should unravel more precise mechanisms by which butyrate-activating PI3K/AKT pathway contributes to CRC drug resistance. There is also a need to explore strategies to optimize CRC treatment outcomes by modulating the gut microbiota, potentially involving targeted interventions that harness the metabolic byproducts of these bacteria. Research should explore the potential synergistic effects of combining PI3K/AKT pathway inhibitors with existing chemotherapeutic regimens.

The identification of the intratumor microbiome and cmDNA signatures as biomarkers of resistance in CRC patients opened avenues for the development of novel therapeutic strategies. Our findings suggested that targeting the molecular pathways associated with butyrate-producing bacteria could be a potential avenue for developing targeted therapies. By modulating the gut microbiota or its metabolites, such as butyrate, we might be able to enhance the efficacy of existing chemotherapies or develop new treatments that are more personalized and effective for CRC patients. Future research should explore these pathways and their interactions with conventional chemotherapy to optimize treatment outcomes. Furthermore, our results indicated that the biomarkers identified in this study could serve as predictive tools for chemotherapy responses in CRC patients. The ability to predict which patients were more likely to respond to specific chemotherapy regimens could greatly inform treatment decisions, allowing for more personalized approaches and potentially improving patient outcomes. Larger studies were needed to validate these biomarkers and to explore their predictive value in various patient populations and treatment settings.

While our study contributed to the body of research exploring the relationship between the gut microbiome and CRC, it built upon previous work published on PubMed. For instance, studies have emphasized the link between gut microbiota dysbiosis and the development of CRC (Li et al., 2024c; Chen et al., 2020). However, our research offered a novel perspective by revealing the role of specific microbial metabolites, such as butyrate, in CRC treatment. Notably, we found that butyrate enhances chemoresistance in CRC cells by activating the PI3K/AKT pathway, different from previous studies. Furthermore, our study aligned with previous studies in observing a reduction in gut microbiota diversity among CRC patients (Li et al., 2024c), but we provided a deeper understanding of the structural and functional changes in the microbial community through 16S rRNA sequencing and cmDNA analysis. We also identified butyrate-producing bacteria, such as Roseburia, as significantly associated with CRC chemoresistance, providing direct experimental evidence that was not implied before. Compared to previous studies, our study not only focused on the impact of the gut microbiome on CRC development but also highlighted its role in chemoresistance. We validated the effects of butyrate on CRC cell proliferation and migration through *in vitro* and *in vivo* experiments, including cell cultures and animal models, which were not extensively investigated before.

There exist some limitations in this study. Firstly, the sample size, while adequate for our analytical approach, was modest, which might limit the power of our findings and their generalizability to other populations. Future studies with larger cohorts would be necessary to confirm our results and to explore potential interactions and effects within different demographic and clinical subgroups. Selection bias was minimized by applying stringent inclusion and exclusion criteria, but this approach might have inadvertently favored specific subgroups, potentially limiting the generalizability of our findings. Moreover, measurement bias was a consideration, as our study relied on exogenous butyrate supplementation to evaluate chemoresistance, which may not accurately represent the endogenous butyrate levels within the tumor microenvironment. This could have influenced the accuracy of our conclusions regarding butyrate’s impact on chemoresistance. It is imperative to acknowledge that the concentration of butyrate within the tumor microenvironment may fluctuate and could pose challenges for precise quantification. While we have controlled for several known confounding factors, there may be other unmeasured variables that could influence the relationship between the microbiome and chemoresistance. The measurement of butyrate and other microbial metabolites in the tumor microenvironment was complex, and our study relied on exogenous supplementation to assess the impact of butyrate on chemoresistance. Future studies employing more precise methods to measure endogenous butyrate levels would be crucial to advance our understanding of its role in CRC. Other bacteria not reaching statistical significance may be attributed to various factors, including sample size limitations, individual patient variations, and differing therapeutic regimens. Future studies would benefit from employing innovative targeted metabolomics methodologies to more accurately assess the dynamic levels of butyrate and other microbial metabolites.

To sum up, this study not only monitored changes in overall bacterial abundance but also delved into specific microbial groups meticulously, elucidating their distinct contributions to CRC drug resistance. Additionally, cmDNA signatures were harnessed for the precise quantification of target microbial abundances, complemented by butyrate administration for CRC resistance evaluation. Significance of this study is anchored in its inaugural systematic comparison of the gut microbiota profiles between CRC patients with and without drug resistance, with a particular emphasis on the quantitative analysis of elevated Fusobacteria and Roseburia. By dissecting the intricate role of the gut microbiota in tumor drug resistance, we pave the way for the development of microbiota-based diagnostic tools and therapeutic strategies. These advancements hold the potential to significantly enhance patient prognosis and quality of life, offering novel insights and targets for chemoresistance. Insights into the intricate interplay between the gut microbiome and CRC highlight potential avenues for developing targeted therapeutic interventions and underscore the utility of microbiome-based biomarkers in the prognostication and treatment of CRC.

## 5 Conclusion

Our investigation identified the intratumor microbiome and cmDNA signatures are promising biomarkers in determining resistance in CRC patients. The dysbiosis of butyrate-producing bacteria, notably within the tumor microenvironment, significantly contribute to the development of tumor resistance. Furthermore, our results suggest that butyrate promote drug resistance through activating PI3K/AKT pathway. Future research should focus on translating these biomarkers into clinical applications. This includes further validation of the intratumor microbiome and cmDNA signatures in larger cohorts to establish their predictive value in personalized treatment strategies.

## Data Availability

The original contributions presented in the study are included in the article/supplementary material, further inquiries can be directed to the corresponding author.
